# Production and quality evaluation of a novel γ-aminobutyric acid-enriched yogurt

**DOI:** 10.3389/fnut.2024.1404743

**Published:** 2024-05-09

**Authors:** Fei Zhu, Sheng Hu, Lehe Mei

**Affiliations:** ^1^Department of Food Science, Zhejiang Pharmaceutical University, Ningbo, China; ^2^Country School of Biological and Chemical Engineering, NingboTech University, Ningbo, China; ^3^Jinhua Advanced Research Institute, Jinhua, China; ^4^College of Chemical and Biochemical Engineering, Zhejiang University, Hangzhou, China

**Keywords:** Levilactobacillus brevis, γ-aminobutyric acid (GABA), yogurt, functional food, *Streptococcus thermophilus*

## Abstract

**Objective:**

γ-aminobutyric acid (GABA) is a neurotransmitter inhibitor that has beneficial effects on various health conditions such as hypertension, cognitive dysfunction, and anxiety. In this study, we investigated a novel yogurt naturally enriched with GABA using a *Levilactobacillus brevis* strain isolated in our laboratory; the specific optimum yogurt production conditions for this strain were determined.

**Methods:**

We isolated an *L. brevis* strain and used it to produce yogurt naturally enriched with GABA. We explored the optimal conditions to enhance GABA yield, including fermentation temperature, inoculation amount, L-monosodium glutamate (L-MSG) concentration, fermentation time, and sucrose content. We also performed mixed fermentation with *Streptococcus thermophilus* and evaluated the quality of the yogurt.

**Results:**

Following optimization (43°C, 8% inoculation amount, 1.5 g/L L-MSG, and 8% sucrose for 40 h of fermentation), the GABA yield of the yogurt increased by 2.2 times, reaching 75.3 mg/100 g. Mixed fermentation with *S. thermophilus* demonstrated favorable results, achieving a GABA yield akin to that found in some commercially available functional foods. Moreover, the viable microbe count in the GABA-enriched yogurt exceeded 1 × 10^8^ cfu/mL, which is higher than that of commercial standards. The yogurt also exhibited a suitable water-holding capacity, viscosity, 3-week storage time, and favorable sensory test results.

**Conclusion:**

This study highlights the potential of naturally enriched GABA yogurt as a competitive commercial yogurt with beneficial health effects.

## 1 Introduction

Health has become an increased focus in many people’s lives because of the COVID-19 pandemic. Studies have reported that severe or fatal outcomes among patients with hypertension risk have increased since 2020 (1–4). Furthermore, the WHO reports an increase in the proportion of relational “sub-health” symptoms with potential lifestyle changes after the pandemic, including sleep problems, cognitive dysfunction, fatigue, low immunity, and obesity (5–7). Notably, many of the reported symptoms are related to diet.

In a concerted action, European Union experts described functional foods as those that have been demonstrated to beneficially affect one or more target functions in the body, relevant to either an improved state of health and well-being and/or a reduction of risk of disease (8). Functional foods could potentially offer a solution to improve above symptoms. Yogurt, a traditional food with reported health benefits, has been included in nearly every national dietary guideline (9–12). Yogurt is easily digestible and particularly suitable for lactose intolerant individuals as it is rich in amino acids, and its lactose is converted to lactic acid. Yogurt also contains diverse peptides related to glucose regulation, lipid storage, and obesity treatment (13, 14), as well as abundant whey proteins characterized by the highest antioxidant potential among all proteins within food products (15). Notably, some microbes found in fermented dairy foods survive digestion in the gastrointestinal tract and help maintain microbial balance (16–18). In addition to the microbes, the active substances produced during fermentation, especially those from microbes naturally existing in the human body, can be beneficial and help to maintain physiological activities. Yogurt functions are differentiated by various active substances. Functional yogurts with plant-sourced components from rice, pepper, and papaya reportedly have a low glycemic index, low fat content, and metabolism-related benefits (19–21). Meanwhile, yogurt fermented from unconventional media, such as goat milk, can provide functional flavor substances (22). Benefits related to intestinal barrier dysfunction, osteoporosis, and hyperglycemia have also been reported for yogurt containing different active substances (23, 24). Although functional yogurt studies have been performed for various molecules, active amino acids represent the most absorbable functional substances for the human body.

γ-aminobutyric acid (GABA) is a non-protein amino acid and a major neurotransmitter involved in rapid inhibitory synaptic transmissions that naturally occur in the central nervous system of mammalian brains (25–28). Inadequate levels of GABA lead to primary insomnia and potential depressive disorders (29, 30), and consequently, GABA intake could help improve sleep quality (31, 32). In addition, GABA is associated with motor learning behavior and learning-related activities in the human brain and is utilized for neuromodulation cascading improvement treatment in individuals (33, 34). Furthermore, GABA is recognized as a beneficial food for blood pressure control, immunity improvement, and weight loss (35–37). Therefore, yogurt rich in GABA could be an ideal and safe treatment method for sub-health.

Although GABA can be chemically synthesized, during the associated processes, it becomes mixed with isomers and by-products, which are costly to thoroughly purify and unsafe as an ingredient for food-grade products. Moreover, although GABA extracted from food, such as rice, beans, and tea, is safe, it is constrained by scale because the GABA content in each food is limited, and plants have long growth cycles (38–40). Both production methods face common challenges as the purification and homogeneity procedures from “GABA compound production” to “GABA yogurt” are issues that must be solved and require extra cost. The direct addition of GABA to milk can result in a large number of whey deposits and influence yogurt texture (41). Microorganisms, especially those separated from foods, could be used as safe and affordable ways to produce GABA. While GABA is produced by different lactic acid bacteria (LAB) (42–45), most studies on GABA microbial cultivation have focused on glucose yeast extract peptone medium or DeMan, Rogosa, and Sharpe medium (MRS) fermentation, and little attention has been paid to milk medium fermentation. Consequently, GABA fermentation directly by LAB in milk remains a promising method for achieving GABA-enriched yogurt, warranting further investigation.

In addition, the quantity of GABA and the quality of yogurt are affected by multiple factors. The fermentation medium is essential for yogurt quality (46–48), and fermentation temperature and time can influence GABA yield (49, 50). Other factors, such as the sources of LAB, can also impact the GABA output of yogurt (51). In the present study, we produced a GABA-enriched yogurt using a *Levilactobacillus brevis* strain isolated in our laboratory. We then investigated and optimized multiple production factors while assessing the quality and physical properties of the yogurt. Consequently, we determined the optimum fermentation parameters for competitive GABA-enriched yogurt production.

## 2 Materials and methods

### 2.1 Materials

Milk powder was purchased from Nestlé Co., Ltd. (Beijing, China), and sucrose was purchased from Taikoo Sugar Co., Ltd. (Shanghai, China). MRS medium, M17 medium, L-monosodium glutamate (L-MSG), and agar were acquired from Sangon Biotech Co., Ltd. (Shanghai, China). Chromatography-grade methanol was acquired from Oceanpak Alexative Chemical Ltd., (Goteborg, Sweden). Sodium acetate and tetrahydrofuran were purchased from Sinopharm Chemical Reagent Co., Ltd. (Shanghai, China). GABA-producing *L. brevis* CGMCC 1306 was isolated from fresh milk in our laboratory. *Streptococcus thermophilus* ATCC 14485 was purchased from the Guangdong Microbiological Culture Collection Center.

### 2.2 Methods

*L. brevis* CGMCC 1306 was isolated from fresh milk in our laboratory and was found to produce GABA. The methods in this study is appropriate for strain culture and GABA determination. Yogurt making and quality evaluation methods are also included in this study.

#### 2.2.1 Bacterial culture

*L. brevis* CGMCC 1306 strain was streaked on fresh MRS agar plates for single colony growth, transferred to MRS broth, and grown anaerobically at 37°C for 48 h. *Streptococcus thermophilus* ATCC 14485 was transferred from commercial agar plates to M17 medium and grown anaerobically at 37°C for 48 h. Agar (1.8% w/v) was added to the corresponding medium if a solid medium was required.

#### 2.2.2 Enumeration of viable cells in yogurt

The method used was based on national food safety standards with modifications (52). A 25 g yogurt sample was mixed with 225 mL of normal saline for dilution. Next, 10 mL of the mixture was added to 90 mL of normal saline, and this step gradient was continued for normal dilution. The MRS agar plates were spread with 100 μL of the corresponding diluents and anaerobically grown at 37°C for 48 h. Single colony numbers were then obtained, and the unit of viable count was described as cfu/mL.

#### 2.2.3 Preparation of GABA-enriched yogurt and multiple factor evaluation

A 10% (w/v) milk powder, with 1.5 g/L L-MSG as a substrate, and 8%(w/v) sucrose were dissolved in sterilized distilled water, homogenized and pasteurized in a water bath at 95°C for 15 min, and then cooled to 43°C. Subsequently, 8% (v/v) *L. brevis* CGMCC 1306 was added to the milk mixture and incubated at 43°C for 40 h. Multiple factors were tested to optimize the GABA yield in the yogurt, and the yogurt was stored at 4°C for 3 weeks continuously to evaluate yogurt storage stability based on commercial yogurt shelf life and storage conditions in the market. The water holding capacity (WHC), viscosity, and GABA stability tests were then conducted. The designed factors were as follows: fermentation time was from 8 to 48 h; fermentation temperature was 31, 34, 37, 40, 43, and 46°C; L-MSG concentration was 0.5, 1.0, 1.5, 2.0, 2.5, and 3.0 g/L; sucrose content was 2, 4, 6, 8, and 10%; and the inoculation amount was 2, 4, 6, 8, and 10%. Mixed fermentation was studied using a 0.25:1, 0.5:1, 1:1, 2:1, 3:1, and 4:1 ratio between *L. brevis* CGMCC 1306 and *S. thermophilus* ATCC 14485.

#### 2.2.4 Determination of GABA content

GABA concentration was determined using high-performance liquid chromatography (HPLC; LC-2030, SHIMADZU, Japan). The sample was pretreated before the HPLC test via centrifugation at 8000 rpm for 3 min (Centrifuge 5,424, Eppendorf, Germany). The supernatant was mixed with 0.5 mol/L NaHCO_3_ and 8 g/L dansyl chloride–acetone solution and then reacted for 1 h at 30°C in the dark. The mixtures were filtered and measured with a chromatographic column (Hypersil ODS2 C18, Elite, China) using a gradient elution procedure with mobile phases A and B. The mobile phase A was ethanol, and mobile phase B was a complex of tetrahydrofuran, methanol, and 0.05 mol/L sodium acetate (5:75:420, v/v/v) (53).

#### 2.2.5 WHC test

The WHC assesses casein coagulate compactness and represents the yogurt texture. The yogurt samples were gently stirred to homogeneity and centrifuged for 10 min at 1250 × *g* at 4°C. The WHC (% w/w) was evaluated by weight change after whey was expelled according to Equation (1) (54) as follows:


(1) $$WHC\;=\;100\left(Y-W\right)/Y$$

where Y is the weight of the yogurt sample, and W is the weight of whey expelled.

#### 2.2.6 Rheological studies in the yogurt

The viscosity of the yogurt was measured at different fermentation periods using a viscometer (DV2T; Brookfield, United States). The samples previously incubated at 4°C were adapted to 25°C for 30 min and fitted with LV-1 rotor at 30 rpm.

#### 2.2.7 Sensory evaluation

The appearance, odor, taste, acidity, texture of the fermented milk, and overall acceptance were estimated by 40 professionally trained panelists, with scores ranging from 1 to 9. The two control groups were commercial yogurts purchased from the market. The quantitative scoring rules are described in Table 1. The method was modified based on Han’s method, and a commercial yogurt was used as a control group (55).

**Table 1:** Sensory evaluation scoring criteria for yogurt.

Item	Yogurt scoring criteria	Score
Appearance	Milky white, glossy, and homogenic	7–9
	Faint yellow, relatively uniform, and glossy	4–6
	Dark and inhomogeneous	1–3
Odor	Obvious pleasant smell that is sustained and no abnormal sour smell	7–9
	Pleasant smell and normal sour smell	4–6
	There is an unpleasant smell	1–3
Taste and acidity	Smooth and soft, sweet and sour balance, not grainy	7–9
	Soft, sour, and slightly grainy	4–6
	Coarse, extremely sour, and unusual taste	1–3
Texture	Uniform and stable, without or with little whey expelled	7–9
	Generally uniform and stable, with a small amount of whey expelled	4–6
	Inhomogeneous and loose, with a large amount of whey expelled	1–3
Overall acceptance	Overall good	7–9
	Acceptable	4–6
	Not recommended to taste	1–3

#### 2.2.8 Statistical analysis

All data are represented as mean ± SD. Data analysis was performed using Origin 8.5. Statistical significance was determined using IBM SPSS Statistics 27. Duncan’s test was used to analyze the statistical significance variability between samples using analysis of variance with a significance level of 95% (α = 0.05). The experiments were conducted in triplicate.

## 3 Results and discussion

### 3.1 Yogurt preparation and factor optimization

#### 3.1.1 Effects of fermentation time on GABA production and microbe growth dynamics

**Figure 1:**
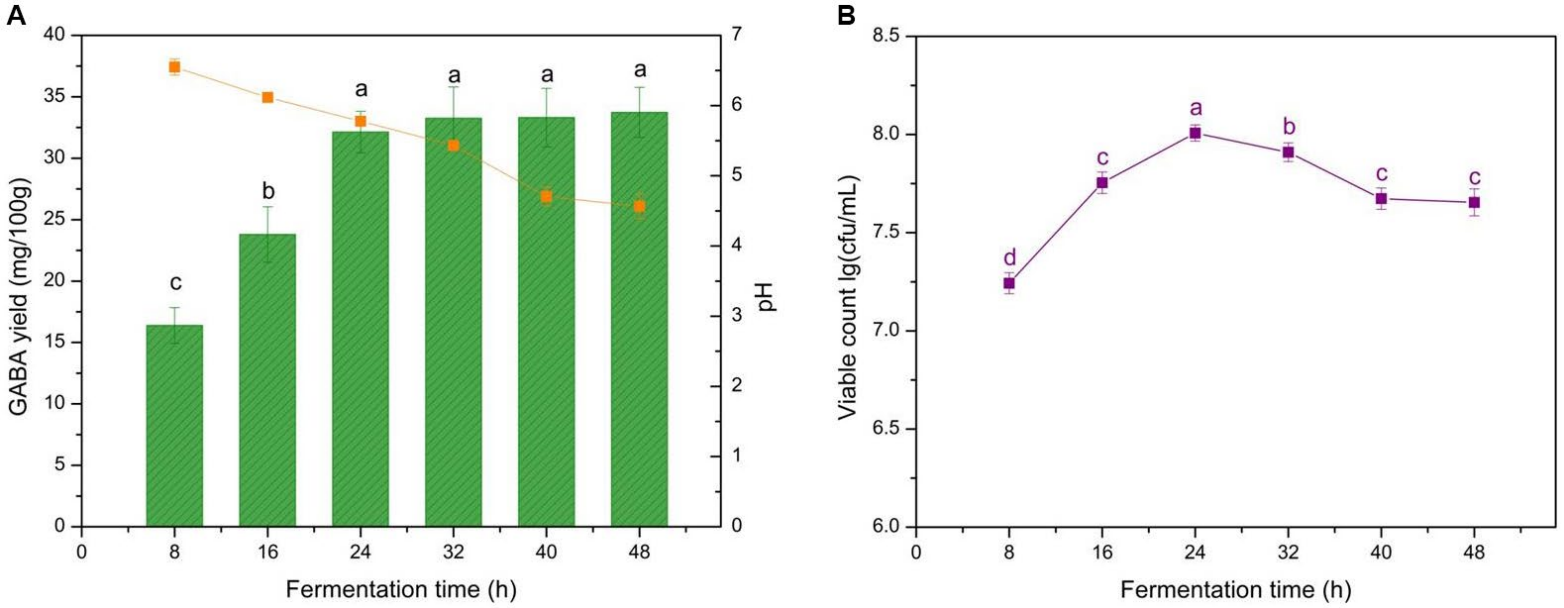
Effect of fermentation time on GABA yield, acidity, and microbial growth in yogurt. **(A)** Effect of fermentation time on GABA yield (histogram) and acidity (orange line). **(B)** Effects of fermentation time on microbial growth (purple line). Data are expressed as the mean ± SD. Different lowercase letters above error bars indicate significant differences (*p* < 0.05, analysis of variance, ANOVA, α = 0.05, Duncan’s test).

Total fermentation time is essential to yogurt production because it determines the microbial growth stage and GABA production. It also influences the yogurt texture as the coagulation level of casein in milk is affected by the acidifying procedure during the fermentation ending point. GABA production, microbe growth dynamics, and acidifying procedure compared with fermentation time are shown in Figure 1. The data of the present study indicated that GABA accumulated continuously in the early stage of fermentation (< 24 h), whereas GABA yield was comparatively stable at 32–40 h with 33.3 mg/100 g. Microbial growth demonstrated a similar trend to GABA production before 24 h, as cells began to decay from 24 h to 48 h. The acidifying procedure was maintained throughout fermentation, and the final yogurt pH decreased to 4.5 at the end of fermentation. Therefore, fermentation time significantly affected GABA yield (*p* < 0.05).

In the early fermentation period, the resources were intensively utilized for biomass increase, and GABA yield was limited. However, it plateaued after 32 h of fermentation as shown in Figure 1A, as the residual microbes aimed to maintain basic physiological activity instead of synthesizing GABA, which led to slight increases in GABA yield. Yogurt pH decreased persistently but slowly owing to the LAB growth, and the final pH was 4.5 shown in Figure 1A. This pH was higher than that of commercial yogurts, which normally ranges from 4.1 to 4.3. This result was attributed to GABA production, which is H^+^ consuming, thus neutralizing the pH decrease resulting from the growth of the LAB. This characteristic could help prevent yogurt over-acidification and, thus, improve shelf life. As expected, *L. brevis* CGMCC 1306 displayed good growth characteristics, even in the initial stage (< 8 h) of fermentation, the viable cell number reached 1.65 × 10^7^ cfu/mL (Figure 1B), which is on par with commercial yogurt. After 24 h, the nutrient levels in the milk gradually decreased and could not support the high level of cell growth; consequently, the number of microorganisms began to decline. Meanwhile, cell growth and decreased pH mutually promote each other. That is, LAB converts the lactose in milk to lactic acid, decreasing the pH and creating a relatively acidic environment to help LAB grow. The procedure stimulated and induced glutamic acid decarboxylase (GAD) activity to gradually catalyze L-MSG to GABA, verifying the GABA accumulation trend. Therefore, 40 h of fermentation was recommended to achieve sufficient GABA yields and an appropriate pH during yogurt production.

#### 3.1.2 Effect of fermentation temperature on GABA production, microbial growth, and pH

**Figure 2:**
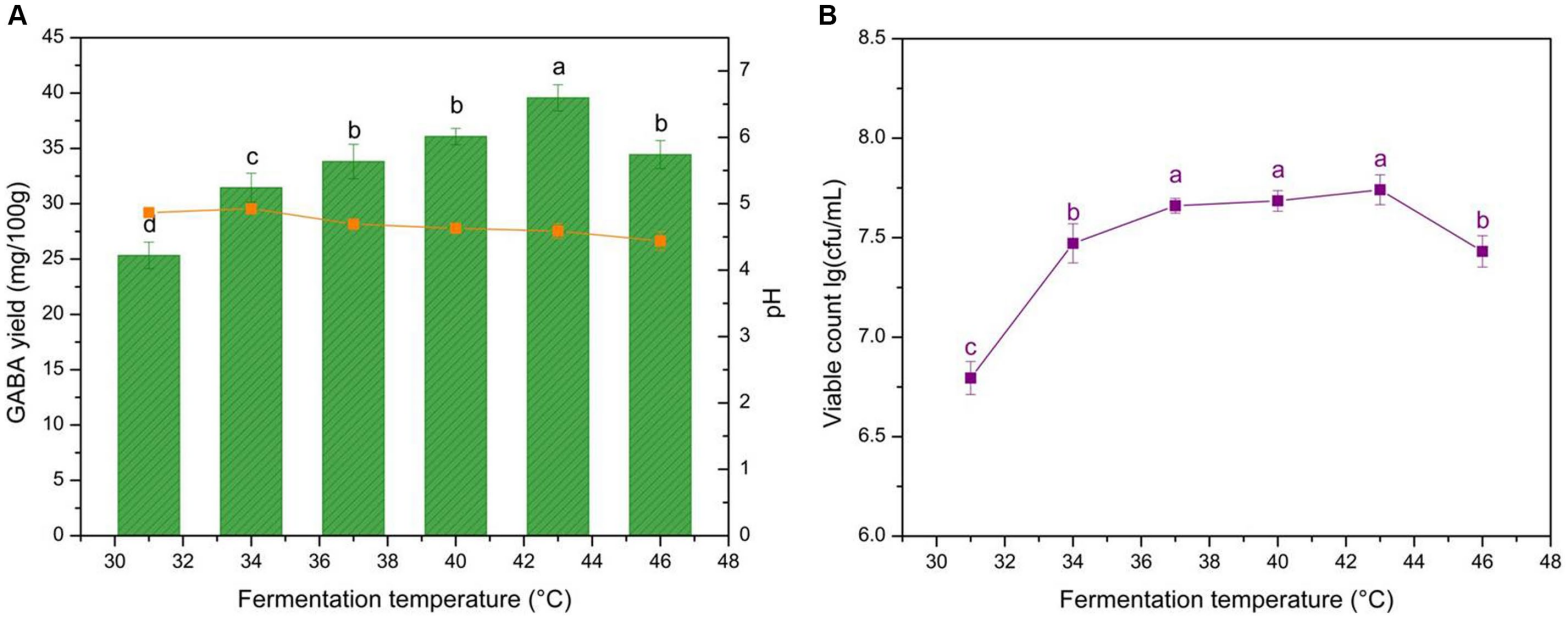
Effects of fermentation temperature on GABA yield, acidity, and microbial growth in yogurt. **(A)** Effect of fermentation temperature on GABA yield (histogram) and acidity (orange line). **(B)** Effect of fermentation temperature on microbial growth (purple line). Data are expressed as the mean ± SD. Different lowercase letters above error bars indicate significant differences (*p* < 0.05; ANOVA, α = 0.05, Duncan’s test).

Fermentation temperature influences microbial growth and exopolysaccharide (EPS) formation, affecting the acidity procedures of different LAB stains, which may influence the yogurt texture and GABA yield (56). The GABA yield, yogurt pH, and microbe growth are shown in Figure 2. GABA yield was the highest at 43°C and microbial growth was limited by high temperature. The pH at the different fermentation temperatures ranged from 4.4 to 4.9. The results indicate that fermentation temperature significantly affected GABA yield (*p* < 0.05).

The GABA yield increased at <43°C and decreased at >43°C (Figure 2A). Two reasons reliably explain the climbing trend. First, the increased temperature aided microbial growth and proliferation. Second, the optimum catalytic temperature for glutamic acid decarboxylase (GAD), which transforms L-MSG into GABA, is >40°C. The higher temperatures caused cell aging and increased the portion of crosslinking between the casein micelle branched chains within the microcavity inner surface of yogurt, reducing the WHC (57, 58). Moreover, the irregular inner structure could lead to the irregular distribution of microbes, potentially increasing GABA mass transfer resistance. This also explains the microbial growth trend (Figure 2B). The final pH is a delicate balance between GABA production which is H^+^ consuming and microbial physiological activity which is H^+^ generating. The influence of fermentation temperature on yogurt pH was not obvious (Figure 2A). The optimal yogurt production temperature selected was 43°C, which is the same as that for commercial yogurt, indicating that GABA-enriched yogurt could potentially be manufactured in dairy plants without additional equipment and energy costs.

#### 3.1.3 Effects of L-MSG on GABA production, microbial growth, and pH

**Figure 3:**
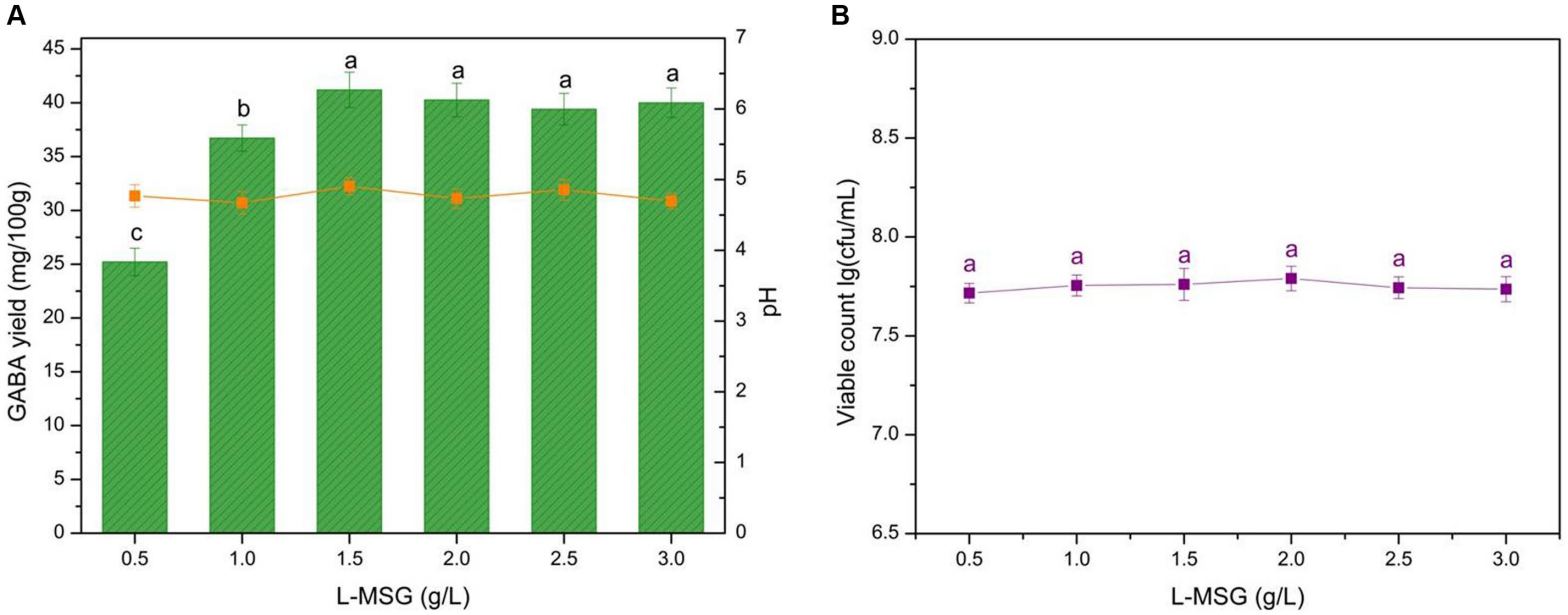
Effect of the L-MSG concentration on GABA yield, acidity, and microbial growth. **(A)** Effect of the L-MSG concentration on GABA yield (histogram) and acidity (orange line). **(B)** Effect of the L-MSG concentration on microbial growth (purple line). Data are expressed as the mean ± SD. Different lowercase letters above error bars indicate significant differences (*p* < 0.05; ANOVA, α = 0.05, Duncan’s test).

L-MSG, a substrate of GAD, can be catalyzed into GABA by LAB. Although some LAB can synthesize GABA using a precursor substance within the cell, the resulting GABA production is limited. The addition of L-MSG into the fermentation liquid is necessary to achieve sufficient GABA production. GABA production, microbial growth, and pH compared with L-MSG concentration are shown in Figure 3. The data indicated that GABA yield increases sharply with an L-MSG concentration of <1.5 g/L, and there is a slight decrease with >1.5 g/L. Therefore, the L-MSG concentration significantly affected GABA yield (*p* < 0.05).

GABA yield exhibited a continuous and rapid increase when the L-MSG addition was <1.5 g/L (Figure 3A). This result was attributed to the abundance of microbes compared with the substrate in the microenvironment, and GAD and microbes could adequately leverage the substrate (Figure 3B). As GABA synthesis precursor, L-MSG could improve carbon flux in the GABA metabolic pathway, thus regulating catalysis. The increase in substrate concentration stimulated a GAD catalysis reaction to move in the forward direction, converting L-MSG to GABA. When the L-MSG addition reached 1.5 g/L, the substrate became excessive compared to GAD. When the GAD is fully loaded, feedback inhibition reduced enzyme activity, resulting in slight decreases in GABA yield (59). Therefore, 1.5 g/L L-MSG was identified as the optimal concentration for yogurt production.

#### 3.1.4 Effects of sucrose on GABA production and pH

**Figure 4:**
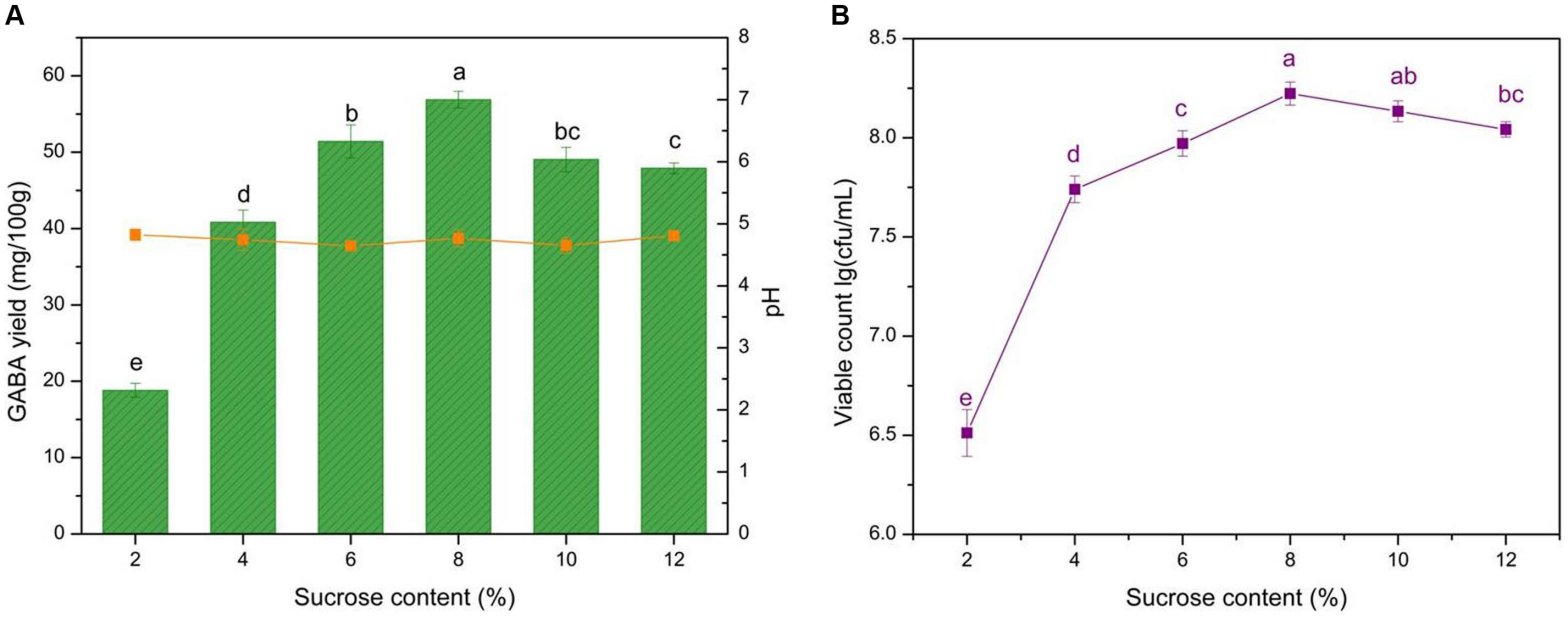
Effect of sucrose content on GABA yield, acidity, and microbial growth. **(A)** Effect of sucrose content on GABA yield (histogram) and acidity (orange line). **(B)** Effect of sucrose content on microbial growth (purple line). Data are expressed as the mean ± SD. Different lowercase letters above error bars indicate significant differences (*p* < 0.05; ANOVA, α = 0.05, Duncan’s test).

Sucrose can be used by microbes as an additional energy source and provides flavor to yogurt. Adding sucrose could potentially improve the robustness and persistence of LAB fermentation. The effects of 2–12% sucrose additions on GABA yield, microbial growth, and pH were analyzed (Figure 4). The data revealed that 6–8% sucrose additions are suitable for GABA production, as the GABA yield reached 56.9 mg/100 g, and microbial growth exhibited a similar trend. Sucrose content significantly affected GABA yield (*p* < 0.05). The exogenous carbohydrate supplement could help LAB growth and GABA production, and this is in line with previous studies (60). Sucrose is an easy-to-use carbohydrate for bacteria that can be decomposed into glucose, readily supplying carbon resources. The addition of sucrose promoted LAB growth, thus complementing the rapid lactose consumption in milk. Although the GABA yield increased notably with 6–8% sucrose additions, it declined with additions exceeding >8%. This could attributed to the inhibition of microbial growth by high osmotic pressure caused by the high sugar concentration. Consequently, 6–8% sucrose additions were deemed optimal for yogurt production.

#### 3.1.5 Effects of inoculation amount on GABA production and pH

**Figure 5:**
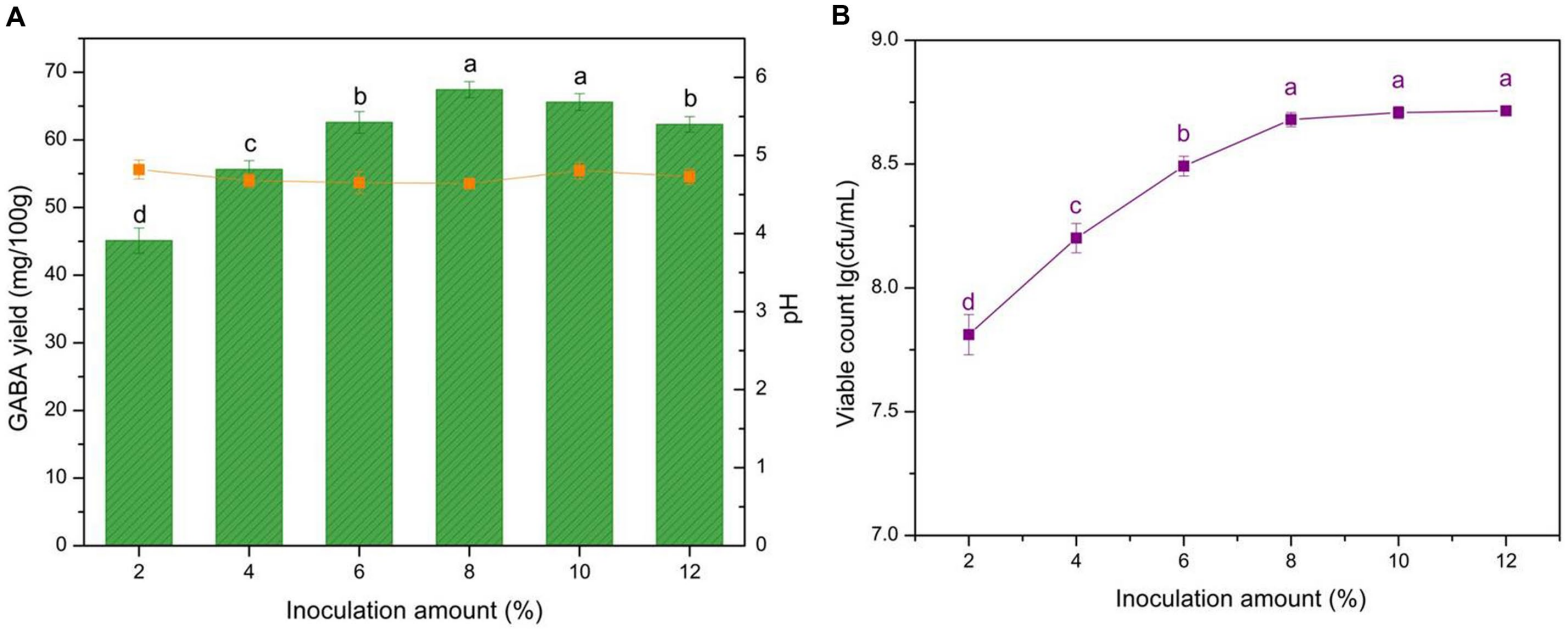
Effect of inoculation amount on GABA yield, acidity, and microbial growth. **(A)** Effect of inoculation on GABA yield (histogram) and acidity (orange line). **(B)** Effect of inoculation on microbe growth (purple line). Data are expressed as the mean ± SD. Different lowercase letters above error bars indicate significant differences of *p* < 0.05 (ANOVA, α = 0.05, Duncan’s test).

The inoculation amount determined the viable microorganism cell numbers during fermentation, particularly within the initial fermentation period. Meanwhile, the viable LAB abundance reflected the GABA production capacity. To determine the optimum microbial quantity for GABA production, different inoculation amounts were evaluated, and microbial growth and acidity were monitored (Figure 5). GABA yield increased with the inoculation amounts, reaching a peak with the 8% addition. The microbial growth increased within the tested range, and the inoculation amount significantly affected GABA yield (*p* < 0.05).

The increase in biomass resulted in notable increases in GABA yield with low inoculation levels (< 8%; Figures 5A,B). This result was attributed to the fact that increasing the inoculation amount could improve the conversion efficiency during this period, with sufficient viable cells leveraging L-MSG and transforming it to GABA. However, when the inoculation amount reached 8%, a slight decrease was observed. This result was attributed to the high cell concentration, which led to L-MSG being outcompeted by the excessive microbes in the microenvironment, hindering its conversion within the inner cells. Moreover, the behavior of the excessive microbes shifted from GABA production to competing for nutrition to ensure survival. Consequently, 8% was selected as the optimum inoculation amount for yogurt production.

#### 3.1.6 Mixed fermentation with *Streptococcus thermophilus*

**Figure 6:**
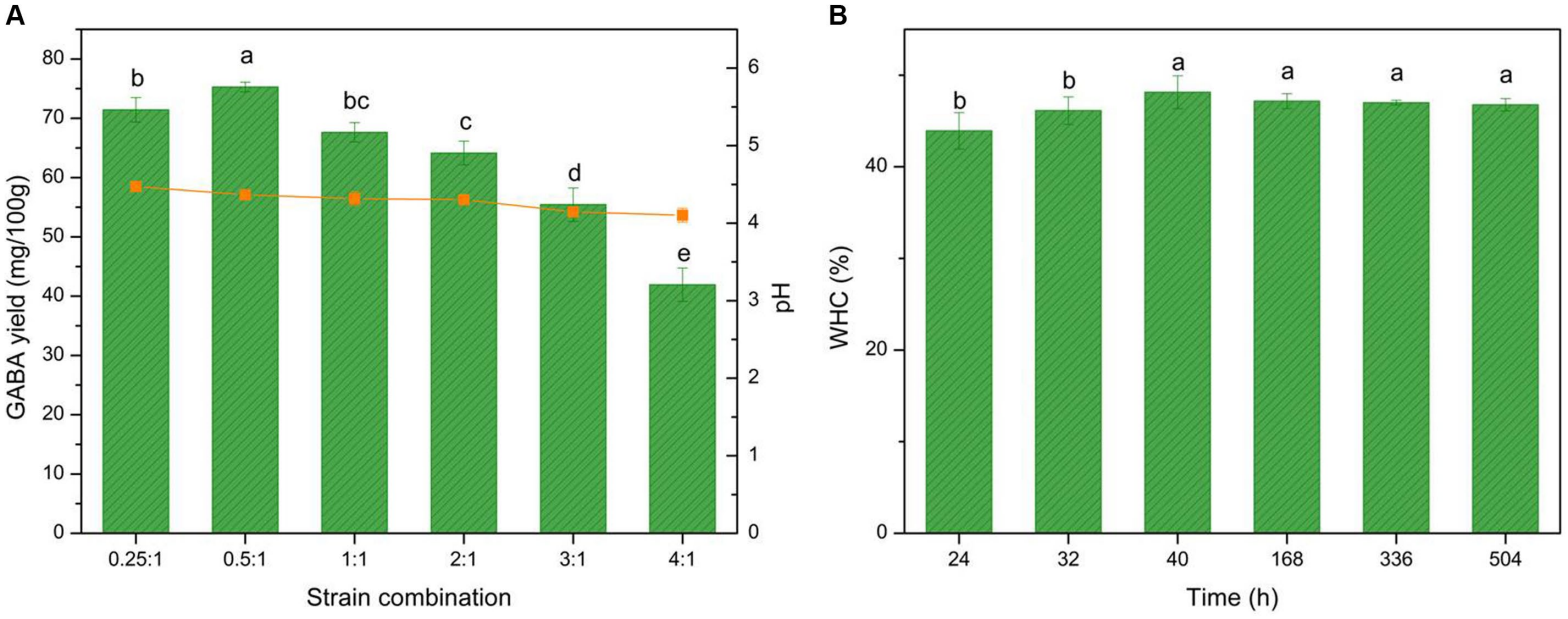
Effect of mixed fermentation on GABA yield, acidity, and water-holding capacity (WHC) curve. **(A)** Effect of mixed fermentation (*S. thermophilus* ATCC 14485 combined with *L. brevis* CGMCC 1306) on GABA yield (histogram), acidity (orange line), and **(B)** the WHC curve during fermentation and storage time. The fermentation period was 24–40 h, and the storage period was 40–504 h. Data are expressed as the mean ± SD. Different lowercase letters above error bars indicate significant differences (*p* < 0.05; ANOVA, α = 0.05, Duncan’s test).

In commercial yogurt, mixed fermentation is often used to improve the sensory and nutritional characteristics of the yogurt. Thus, the GABA production of *L. brevis* CGMCC 1306 under mixed fermentation conditions was evaluated. It was fermented with different proportions of *S. thermophilus* ATCC 14485. The GABA yield and acidity results are shown in Figure 6A. The data demonstrated that *L. brevis* CGMCC 1306 exhibited good GABA production when the ratio between *S. thermophilus* ATCC 14485 and *L. brevis* CGMCC 1306 was 0.5:1. GABA production was significantly enhanced by mixed fermentation, reaching nearly 75.3 mg/100 g. The pH at the fermentation endpoint was 4.1–4.4, which is lower than with pure fermentation. Therefore, mixed fermentation significantly affected GABA yield (*p* < 0.05).

The GABA yield with mixed fermentation may be closely related to the symbiotic effects between *S. thermophilus* and *L. brevis* (61). Ratios of 0.5:1 and 0.25:1 were deemed suitable for GABA production, as the GABA yields achieved maximum values of 75.3 mg/100 g (Figure 6A). At these ratios, the *S. thermophilus* cells grew rapidly and produced formic acid, folic acid, and carbon dioxide. The acidic environment created by *S. thermophilus* cells could accelerate the speed of *L. brevis* growth. Simultaneously, *L. brevis* could massively hydrolyze proteins in milk into peptides or various amino acids, aiding *S. thermophilus* growth and thus promoting GABA production. In this way, the two strains maintained a valuable symbiotic relationship. When the ratio was >1:1, the biomass in the fermentation liquid became too high, and the cells began to compete for the nutrition in the milk, resulting in reduced GABA production. Owing to the strong acidification ability of *S. thermophilus,* the yogurt pH with mixed fermentation was lower than with pure fermentation with one strain at the fermentation ending point. The large, strong casein coagula agglomerated by lower pH also increased the mass transfer resistance for GABA diffusion, which reduced the GABA yield. In conclusion, a ratio between *S. thermophilus* ATCC 14485 and *L. brevis* CGMCC 1306 of 0.5:1 was identified as optimal for GABA-enriched yogurt mixed fermentation.

### 3.2 Yogurt evaluation

#### 3.2.1 WHC

WHC demonstrates the ability of macromolecular substances to combine with water in the yogurt, which indicates the stability of the system. The WHC was tested throughout the fermentation and storage periods for the GABA-enriched yogurt to estimate yogurt syneresis. The relationship between WHC and time is shown in Figure 6B. Throughout the fermentation period, the WHC was affected by fermentation time. However, during the storage period, there were no significant results with time.

The WHC began to increase when casein precipitation was observed, rising from 43.9% at 24 h of fermentation to 48.1% at 40 h of fermentation, and remained relatively stable during the 3-week storage period. With increased fermentation time, LAB growth and the pH of bulk liquid decreased, and the weight of the casein coagulates increased, causing an increase in the WHC. After fermentation, the yogurt was stored at 4°C, and the whey was slightly expelled but remained stable during the storage period (62). The WHC was comparable to that of commercial yogurt, indicating that the GABA-enriched yogurt exhibited good stability.

#### 3.2.2 Viscosity

**Figure 7:**
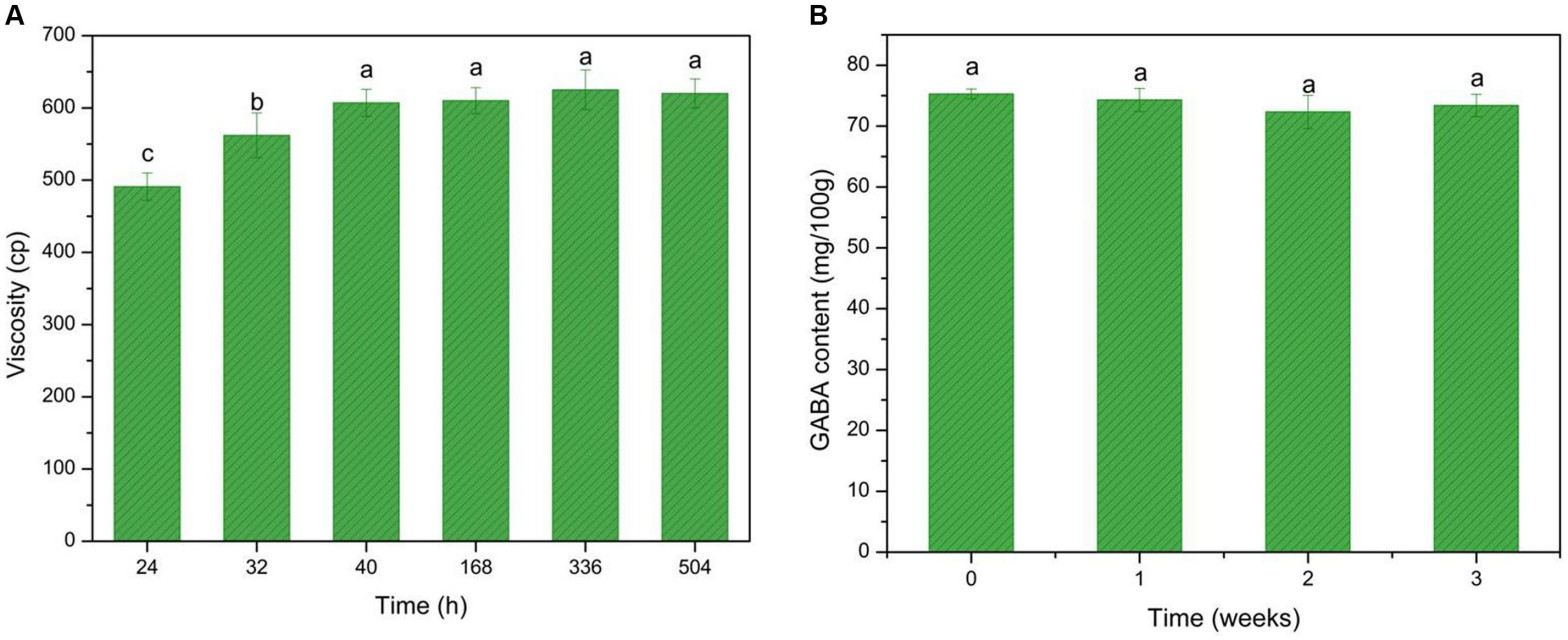
GABA-enriched yogurt consistency and GABA storage stability test. **(A)** GABA viscosity changes during fermentation and the storage period. **(B)** GABA stability test during the storage period. Data are expressed as the mean ± SD. Different lowercase letters above error bars indicate significant differences (*p* < 0.05; ANOVA, α = 0.05, Duncan’s test).

Viscosity is another characterization of the yogurt texture. The casein in milk coagulates due to the acidification process during milk fermentation, which alters yogurt viscosity (Figure 7A). The data showed an increase in viscosity during yogurt fermentation and storage time, ultimately reaching 620 cp.

The coagulum in the yogurt consisted mainly of casein, and the connections between coagulated casein contributed to the increased viscosity. Moreover, EPS produced by microbes during fermentation also contributed to the increased viscosity. The viscosity of the GABA-enriched yogurt was comparable to that of some commercially available yogurts and showed stability during the storage period.

#### 3.2.3 Sensory assessment of the GABA-enriched yogurt

Sensory tests can provide an overall description of a yogurt. Appearance, odor, taste, acidity, texture, and overall acceptance were tested in this study (Table 2).

**Table 2:** Sensory test results for GABA-enriched and commercial yogurts.

Item	GABA-enriched yogurt	Commercial yogurt 1	Commercial yogurt 2
Appearance	8.23 ± 0.20^a^	8.24 ± 0.15^a^	8.30 ± 0.24^a^
Odor	7.85 ± 0.42^a^	7.95 ± 0.34^a^	7.82 ± 0.37^a^
Tasty and Acidity	7.38 ± 0.23^b^	7.66 ± 0.19^a^	7.68 ± 0.20^a^
Texture	8.12 ± 0.31^a^	8.11 ± 0.36^a^	8.15 ± 0.31^a^
Overall Acceptance	7.90 ± 0.24^a^	8.02 ± 0.34^a^	7.96 ± 0.30^a^

Data are expressed as the mean ± SD. Different lowercase letters at the upper right corner of the numbers indicate significant differences (*p* < 0.05, ANOVA, α = 0.05, Duncan’s test).

The data showed that overall acceptance was not significantly different between GABA-enriched yogurt and commercial yogurt. The appearances and textures of the three types of yogurt tested received similar rankings. The odor of commercial yogurt was slightly better than that of GABA yogurt, but not significantly. The taste and acidity of the two commercial yogurts were rated equally and were considered better than those of the GABA-enriched yogurt. The chosen commercial yogurts were common and popular in the market. These results indicated that GABA-enriched yogurt was competitive with commercial yogurt in terms of its sensory characteristics, and it is expected that the health benefits conferred by GABA would further improve its ranking.

#### 3.2.4 GABA storage stability

The storage stability of the GABA-enriched yogurt was tested (Figure 7B). The data indicated robust stability, as during the 3-week storage period, the GABA content was maintained at 75 mg/100 g. This provides further evidence of the robustness of the GABA-enriched yogurt.

Overall, the target yogurt with a pH of 4.37 ± 0.06 and acidity 89 ± 2 °T, fermented with naturally accumulated GABA, exhibited competitive sensory quality and stability compared to commercial yogurt. This yogurt might be effective for improving sleep quality, hypertension, and anxiety. Moreover, the potential applications for GABA-enriched yogurt could be expanded, given its inability to develop drug resistance.

The GABA-enriched yogurt provides a comprehensive nutrition product for the dairy industry. This novel yogurt provides triple benefits for human health: viable LAB benefits intestinal health, GABA provides additional functions, and the yogurt is nutrient-rich. Moreover, for traditional dairy manufacturers, the optimized yogurt production condition described in this study could be achieved using existing equipment without extra processing investment. The only additional material required for fermentation compared with conventional yogurt is L-MSG, the cost of which is negligible compared with other materials. Furthermore, GABA-enriched yogurt as a new competitive functional food with broad applications could provide manufacturers with a wider range for independent pricing to achieve optimum returns.

In this study, mixed fermentation was beneficial to GABA production, suggesting an opportunity for further GABA yield increases in future investigations. In addition, mixed fermentation could improve the taste of yogurt by increasing the flavor substances under the symbiotic effects between two or even three strains. Furthermore, additional sugar resources could be considered as extra energy sources for LAB cell growth and GABA production since sucrose has proven beneficial for GABA yield.

## 4 Conclusion

In this study, a novel GABA-enriched yogurt fermented by a new strain *L. brevis* CGMCC 1306 was developed using multiple factors and evaluated. The yogurt naturally gathered GABA during fermentation without extra cost from processing, and specific production condition for this strain was determined. Factors such as fermentation time, temperature, substrate concentration, sucrose level, and inoculation amount were assessed and found to significantly influence the GABA yield. Moreover, mixed fermentation was investigated to explore the symbiosis between the target LAB and *S. thermophilus*. Optimal fermentation conditions were determined to be a fermentation time of 40 h, at 43°C, with 8% inoculation, 1.5 g/L L-MSG, and 8% sucrose. Mixed fermentation of LAB and *S. thermophilus* at a 0.5:1 ratio maximized symbiosis and effectively stimulated GABA production, resulting in a GABA yield of 75.3 mg/100 g. The yield doubled compared to the original GABA yield prior to optimization and exceeded the effective dose per 100 g of yogurt. The resulting yield was in the same range as that of some commercially available GABA-enriched functional food products (35). The viable count in yogurt was over 1 × 10^8^ cfu/mL, which meets the commercial standard. The yogurt was also found to exhibit suitable characteristics, including those related to taste and storage stability. These results provide a solid foundation for the manufacturing of naturally GABA-enriched yogurt. A feasibility study related to yogurt additives and the behavior of GABA-enriched yogurt on human health should be further considered in the scope of future research.

## Data availability statement

The original contributions presented in the study are included in the article/supplementary material, further inquiries can be directed to the corresponding author.

## Author contributions

FZ: Conceptualization, Data curation, Formal analysis, Methodology, Writing – original draft. SH: Validation, Writing – review & editing. LM: Writing – review & editing.
